# Barracuda: a dynamic, Turing-complete GPU virtual machine for high-performance simulations

**DOI:** 10.1007/s11517-025-03438-3

**Published:** 2025-09-13

**Authors:** Phillip Duncan-Gelder, Darin O’Keeffe, Philip J. Bones, Steven Marsh

**Affiliations:** 1 School of Physical and Chemical Sciences, University of Canterbury, Christchurch, New Zealand; 2 Te Whatu Ora - Health New Zealand, New Zealand, https://www.tewhatuora.govt.nz; 3 Electrical and Computer Engineering, University of Canterbury, Christchurch, New Zealand

**Keywords:** Biomedical simulations, MRI Simulation, CUDA, GPU virtual machine, Magnetic resonance imaging, Medical imaging simulation, GPU acceleration

## Abstract

**Abstract:**

Accurate simulation of dynamic biological phenomena, such as tissue response and disease progression, is crucial in biomedical research and diagnostics. Traditional GPU-based simulation frameworks, typically static CUDA^®^ environments, struggle with dynamically evolving parameters, limiting flexibility and clinical applicability. We introduce Barracuda, an open-source, lightweight, header-only, Turing-complete virtual machine designed for seamless integration into GPU environments. Barracuda enables real-time parameter perturbations through an expressive instruction set and operations library, implemented in a compact C/CUDA library. A dedicated high-level programming language and Rust-based compiler enhance accessibility, allowing straightforward integration into biomedical simulation workflows. Benchmark validations, including Rule 110 cellular automaton and Mandelbrot computations, confirm Barracuda’s versatility and computational completeness. In magnetic resonance imaging (MRI) simulations, Barracuda allows for the dynamic recalculation of critical parameters, such as $T_1$ relaxation times and temperature-induced off-resonance frequencies. Although it introduces computational overhead compared to static kernels, Barracuda significantly improves simulation accuracy by enabling dynamic modeling of key biological processes. Barracuda’s modular architecture supports incremental integration, providing valuable flexibility for biomedical research and rapid prototyping. Future developments aim to optimize performance and expand domain-specific instruction sets, reinforcing Barracuda’s role in bridging static GPU programming and dynamic simulation requirements.

**Graphic abstract:**

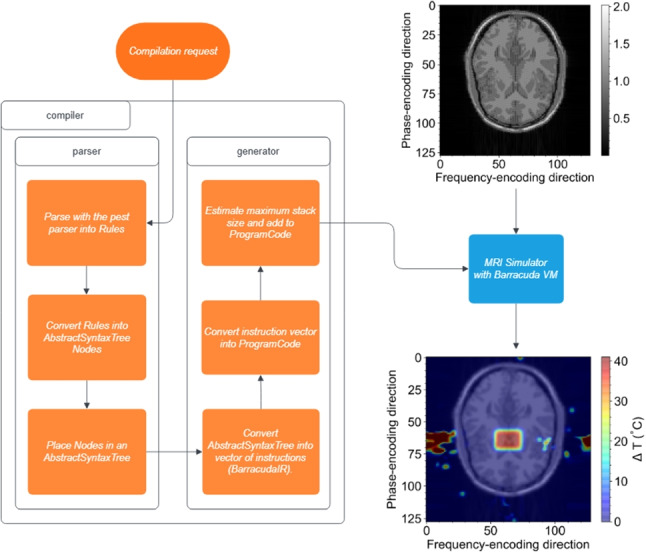

## Introduction

Advances in GPU architectures have revolutionized scientific simulation, enabling researchers to address increasingly complex challenges across diverse fields such as biomedical imaging, computational fluid dynamics, materials science, and financial modeling. Enhanced computational capability has significantly accelerated simulation runtimes and expanded the scope of phenomena that can be accurately modeled. However, as simulations grow in complexity, there is a parallel increase in the necessity for dynamic adaptability. Many applications require real-time parameter adjustments to capture transient phenomena or respond to evolving conditions. For example, in magnetic resonance imaging (MRI) simulations, parameters like tissue relaxation times, RF pulse characteristics, and magnetic field inhomogeneities [1] often exhibit spatial and temporal variability, necessitating dynamic recalibration during simulation.

Traditional GPU programming frameworks such as CUDA [2–4] and OpenCL [5] primarily rely on static compilation, fixing simulation parameters at kernel launch. While highly optimized, this approach inherently lacks the flexibility required for dynamically evolving scenarios typical of biomedical simulations. Dynamic biological processes, such as contrast agent diffusion or thermal tissue effects, demand runtime adaptability that static GPU frameworks cannot effectively provide.

We introduce Barracuda, an open-source, lightweight, header-only (easy to incorporate), Turing-complete virtual machine explicitly designed for high-performance GPU computing to bridge this critical gap. Barracuda compiles high-level simulation code into optimized bytecode [6, 7], establishing a dynamic runtime environment that supports real-time parameter modifications without incurring significant memory or computational overhead. Inspired by a reverse polish notation (RPN)–based virtual machine used for genetic programming [8], Barracuda significantly extends these concepts by incorporating robust control flow mechanisms, advanced data structures, and a streamlined yet expressive instruction set tailored for GPU execution.

Implemented as a compact C/CUDA library, Barracuda seamlessly integrates into existing CUDA-based simulation workflows. Accompanied by a dedicated high-level programming language and Rust-based compiler, it abstracts away bytecode complexities, empowering users to efficiently manage dynamic parameter adjustments without extensive modification to validated GPU code. While our primary application examples are drawn from biomedical MRI simulations demonstrating dynamic recalculation of $T_1$ relaxation times [1] and temperature-driven off-resonance frequency modulation [1, 9], Barracuda’s architecture remains inherently general purpose and applicable to any GPU-based simulation requiring runtime adaptability.

While other high-level abstraction layers (e.g., Thrust, LLNL RAJA, Alpaka) [10–12] and language transformation frameworks exist (e.g., JAX, Numba, CuPy) [13–15], these primarily target just-in-time or ahead-of-time compilation or transpilation of code into CUDA kernels and/or functions. Barracuda does not focus on compiling directly into CUDA kernels or parallel thread execution (PTX) code generation; instead, it compiles to an intermediate bytecode, which is interpreted at runtime. This offers increased flexibility as parameters themselves can be directly modified and shared between a static CUDA interface and the dynamic runtime Barracuda environment. Additionally, this provides increased robustness in terms of handling user inputs for validated simulation codes. Introducing Barracuda into a simulation tool enables user input without requiring direct modification of the underlying simulation code. Therefore, user-defined perturbations are possible without breaking existing validated simulation tools.

This paper details Barracuda’s architectural design, instruction set, and compiler infrastructure and presents experimental evaluations within MRI simulation frameworks. Our work highlights Barracuda’s broad potential for dynamic GPU-based simulations, effectively balancing the high performance of static GPU kernels with the runtime flexibility essential for accurately modeling complex, evolving phenomena.

## Methodology

This section details the design, implementation, and application methods underlying Barracuda. First, we outline Barracuda’s core architectural features and specifications, emphasizing its lightweight and modular design, instruction set, and compiler infrastructure. Subsequently, we describe its integration within MRI simulation scenarios, demonstrating Barracuda’s ability to dynamically adapt key simulation parameters, thereby enhancing the flexibility of biomedical simulations.

**Table 1:** Barracuda virtual machine instructions

Instruction	Description
OP	Execute the operation at the current head of the operation array
VALUE	Push a value onto the stack from the value array
JUMP	Jump to the instruction pointer specified by the top of the stack
JUMPIFZERO	Conditional jump if the top of the stack is zero
SI_VALUE_OP	Single instruction: perform a VALUE followed by an OP
SI_OP_VALUE	Single instruction: perform an OP followed by a VALUE

**Table 2:** Categories and examples of Barracuda virtual machine operations

Category	Example operations
Null/No-Op	OPNULL
Stack access and pointer arithmetic	SREAD, SWRITE, SADD_P, SSUB_P,..
Basic arithmetic and logic	ADD, SUB, MUL, DIV, AND, NOT, SWAP,..
Memory management & Ternary	MALLOC, FREE, MEMCPY, TERNARY,..
Comparison	EQ, GT, LT, NEQ,..
Extra memory Access	PTR_DEREF,..
Floating-point I/O	READ_F32, WRITE_F32,..
Integer I/O	READ_I32, WRITE_I32,..
Character I/O	READ_CHAR, WRITE_CHAR,..
Mathematical functions	ACOS, SIN, SQRT, LOG,..
System calls	PRINTC, PRINTF,..
Special operations	OPINTEGRATE,..
Environment operations	LDPC, LDTID, LDSTK_PTR,..
Type-casting	LONGLONGTODOUBLE, DOUBLETOLONGLONG,..
Synchronization	SYNCWARP, SYNCBLOCK, SYNCGRID,..

### Development and specifications

Barracuda is implemented as a header-only C/CUDA library designed for seamless integration into existing simulation frameworks. Four main objectives guided its development: to provide dynamic flexibility, to incur minimal overhead, to offer extensibility through a minimal yet expressive instruction set, and to allow all this without modification of existing simulation code. At its core, Barracuda is built around a small set of high-level instructions (shown in Table 1) that manage an internal runtime stack and control flow. These instructions include functionality for executing pre-defined operations, pushing constant values onto the stack, and performing unconditional and conditional jumps. They also allow for the combination of common instruction sequences into composite commands. This core instruction set interacts with a library of over 150 operations (illustrated by Table 2) that cover various functions. These include arithmetic (such as addition, subtraction, multiplication, and division), logical operations, memory management (including allocation, deallocation, and memory copying), comparisons, type conversions, and specialized functions such as numerical integration. Barracuda compromises between maintaining low-level hardware control and providing the high-level expressiveness necessary for complex simulation tasks by separating the fundamental control instructions from the extensive operation library.

**Fig. 1:**
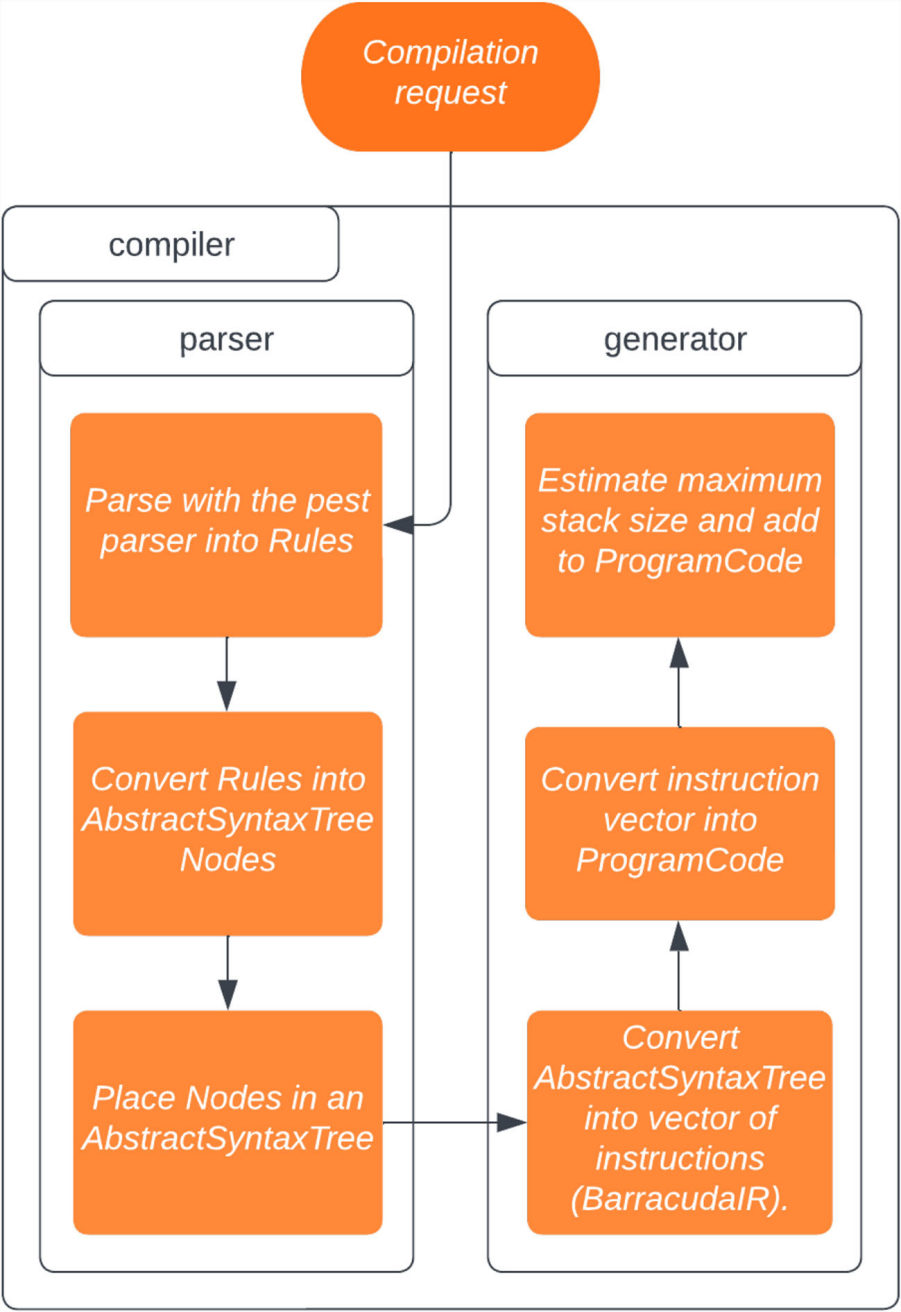
Flow chart of the Barracuda compiler showing the compilation process from high-level source code through to bytecode outputs for the Barracuda runtime

Recognizing that programming directly in low-level bytecode is mentally challenging and, hence, error-prone, we developed a high-level programming language tailored for Barracuda. Accompanied by a dedicated compiler written in Rust [16–18], Barracuda abstracts the complexities of the VM’s bytecode and allows developers to write code using familiar constructs such as arrays, loops, conditionals, and polymorphic function dispatch. To illustrate the mental challenge of writing VM code without the compiler, Appendix A shows both the Rule 110 code and the bytecode arrays (first 50 out of 486 elements shown for illustrative purposes) generated by the compiler.

The compilation process begins with parsing the source code into an Abstract Syntax Tree, followed by semantic analysis for symbol resolution and type checking, and optimizations [19]. This process is illustrated by Fig. 1. The compiler then performs instruction selection and applies further optimizations to produce GPU-targeted bytecode sequences, minimizing redundant operations and improving memory locality. Finally, the compiler returns bytecode arrays, which are loaded (by CPU to GPU transfer, which must be handled and optimized by the host library in which Barracuda is implemented) into the Barracuda VM at runtime. The high-level language reduces the cognitive burden on developers and facilitates rapid prototyping and iterative refinement of simulation routines.

Barracuda can operate both as an embedded virtual machine within existing CUDA applications or as a standalone interpreter. When embedded, a CUDA application invokes Barracuda through an evaluation method, supplying specific instruction, operation, and data arrays (the CPU-GPU transfers of these arrays are all handled by the host application to allow for individual library-level optimizations). This evaluation method executes the Barracuda VM and can modify memory regions designated as accessible “environment variables” provided by the host CUDA application. These modifications enable dynamic interactions between the host application and Barracuda during runtime. A Rust-based compiler facilitates usability by compiling a user-friendly, high-level language into optimized bytecode arrays. The host CUDA application then receives these bytecode arrays and associated instruction and data inputs to determine how the Barracuda VM executes within its simulation framework.

### Applications in MRI simulations

Beyond its general purpose capabilities, Barracuda is integrated into our MRI simulation engine, PhoenixMR [20], enabling the modeling of complex phenomena without requiring direct simulator code modification. This approach achieves a level of flexibility not yet achieved by other generalized simulation frameworks [21–26]. This section shows that by extension of PhoenixMR with Barracuda, we are able to achieve flexibility without directly modifying the validated MRI simulation code, which is currently not yet achievable with the other generalized GPU-based MRI simulators.

This end-to-end approach minimizes the need for extensive modifications to existing CUDA code by passing data, instructions, and operands as simple arrays, allowing dynamic evaluation routines to integrate seamlessly into the simulation process. In our implementation for MRI simulation [20], we solve the Bloch equations using a symmetric Strang splitting method [27], where excitation and relaxation operators are applied sequentially. At each time step, the simulation leverages Barracuda routines to adjust key parameters dynamically, ensuring accurate and efficient simulation. Two sets of Barracuda routines were used—one to modulate $T_1$ relaxation times, simulating the diffusion of a contrast agent, and another to adjust the off-resonance frequency, modeling temperature-related effects. By updating these parameters in real time, the simulation captures dynamic behaviors observed in clinical MRI, thereby enhancing both the realism and adaptability of the model.

The following $T_1$ non-conservative propagator is used to model the diffusion of a contrast agent. Mass conservation is disregarded as we focus on changes in $T_1$ only rather than the fluid mass itself.

1 $$\begin{aligned} P(x,y,t) = \exp \bigg (-\frac{(x-x_0)^2 + (y-y_0)^2}{2(\sigma _0^2 + 2Dt)}\bigg )\exp (-\lambda \rho (x,y)t) \,\,. \end{aligned}$$

Equation 1 describes the spatial and temporal spread of the agent through tissue. This propagator is incorporated into the dynamic recalculation of local $T_1$ relaxation times as given by

2 $$\begin{aligned} T_1(x,y,t) = T_1(x,y,0) - A \cdot T_1(x,y,0) \cdot P(x,y,t) \,\,. \end{aligned}$$

To simulate temperature-induced perturbations in the magnetic field, a heat kernel is also used:

3 $$\begin{aligned} H(x,y,t)= &   \frac{T_0}{4}\bigg ( \big (\text {erf}(\frac{x + 0.5a}{2\sqrt{k_xt}}) - \text {erf}(\frac{x - 0.5a}{2\sqrt{k_xt}})\big ) \big (\text {erf}(\frac{y + 0.5b}{2\sqrt{k_yt}}) \nonumber \\  &   - \text {erf}(\frac{y - 0.5b}{2\sqrt{k_yt}})\big ) \bigg ) \,\,, \end{aligned}$$

and the corresponding off-resonance frequency is calculated according to theory [9, 28] as

4 $$\begin{aligned} \Delta \omega (x,y,t) = \Delta \omega _0 - \alpha \gamma H(x,y,t) \,\,. \end{aligned}$$

Barracuda evaluates these equations in real time, ensuring that changes in $T_1$ and thermal effects on the off-resonance frequency are accurately characterized throughout the simulation.

## Results

This section presents evaluations of Barracuda’s capabilities through benchmark and simulation examples. First, we demonstrate Barracuda’s computational completeness and evaluate its parallel performance using two established benchmarks. We then illustrate its effectiveness in MRI simulations, highlighting Barracuda’s dynamic adaptability for modeling complex, biologically relevant phenomena.

### Benchmark validation

**Fig. 2:**
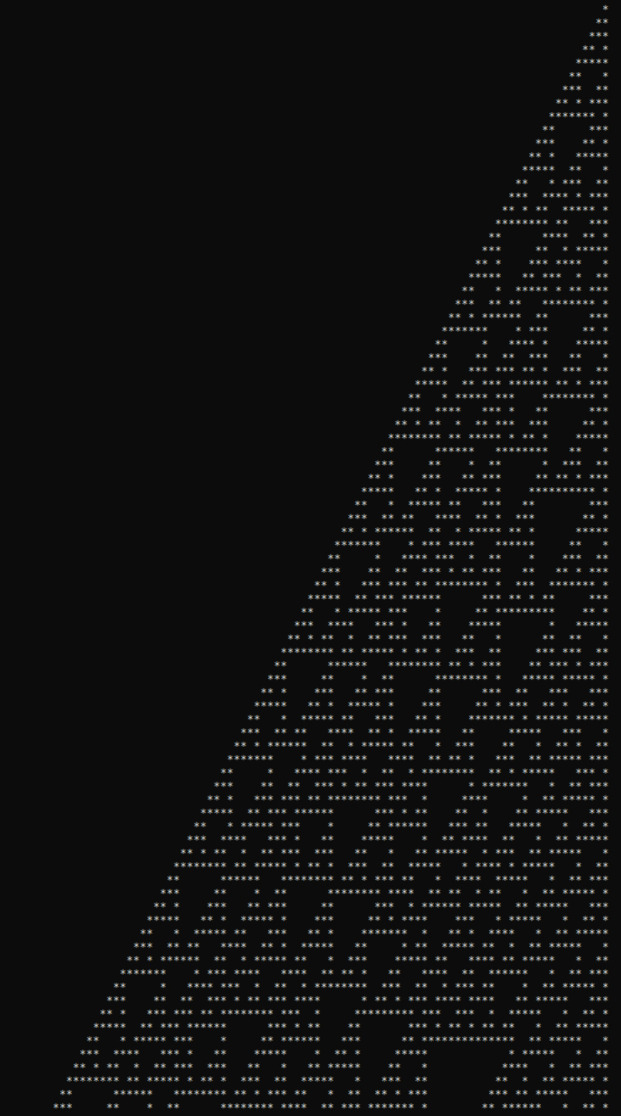
Console output of the Rule 110 program compiled with the Barracuda compiler and run on a standalone Barracuda interpreter. The stars represent values of one, and zeros are represented by empty cells, with each generation being simulated on a new line. The pattern shown correctly reproduces the expected behavior of this automaton [29, 31]

Two benchmarks were selected to validate Barracuda’s computational completeness, flexibility, and parallel performance: the Rule 110 cellular automaton and the Mandelbrot set computation. Rule 110, recognized for its Turing completeness [29, 30], demonstrates Barracuda’s capacity to handle complex logic and intricate control flow effectively. Over 100 generations within a domain of 310 cells, Barracuda’s Rule 110 implementation produced results consistent with theoretical expectations [29, 31] (see Fig. 2), confirming its correctness in executing dynamically evolving computations.

**Fig. 3:**
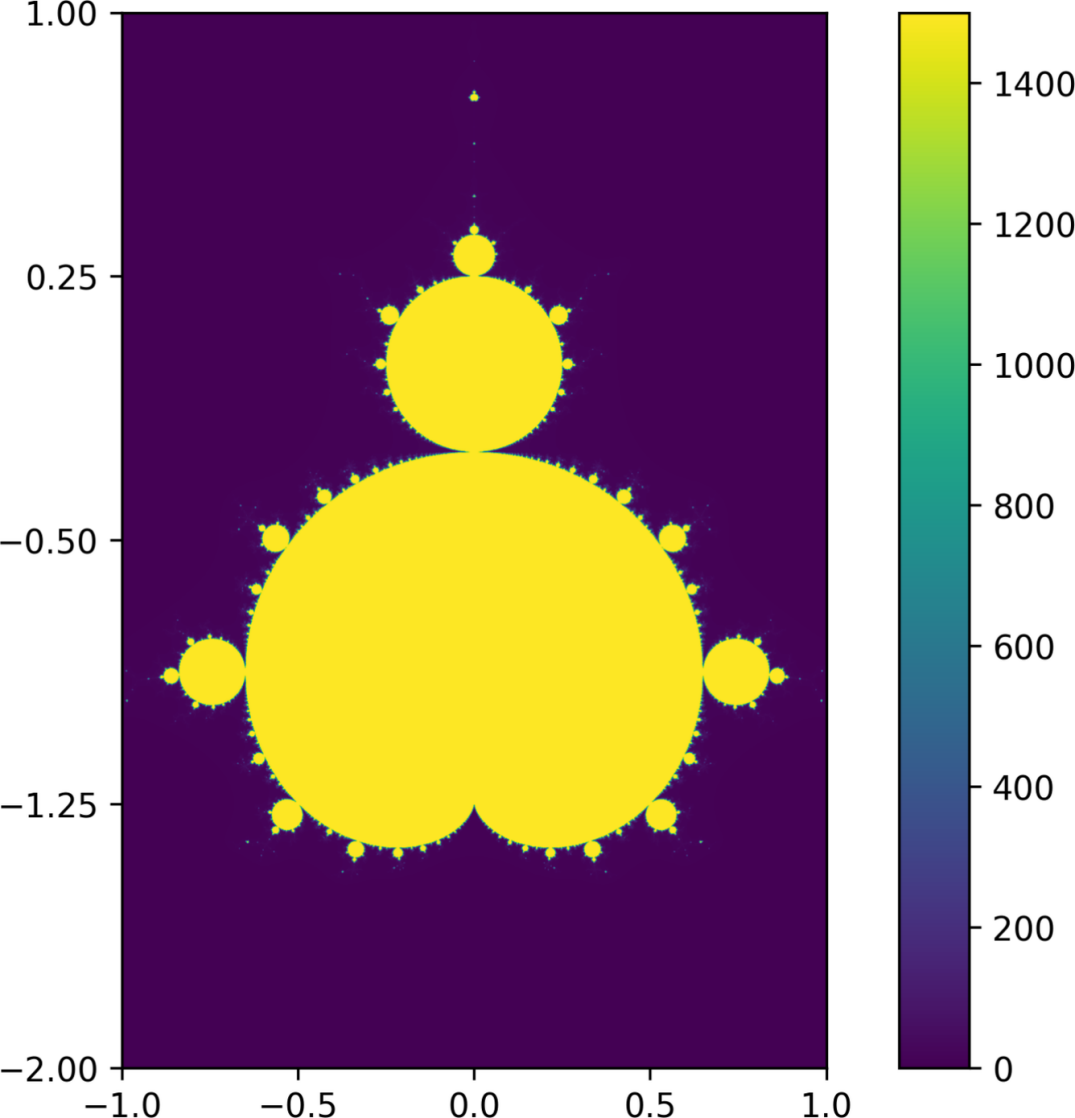
Parallelized Mandelbrot solution compiled with the Barracuda compiler and run on a standalone interpreter. Pixel values represent the number of iterations bounded by an upper limit of 1500 iterations (where values fail to escape with the escape time algorithm [33])

The Mandelbrot set computation, based on the escape time algorithm [32, 33], provides a clear assessment of Barracuda’s parallel performance. Each GPU thread independently computed pixel values to render the Mandelbrot set, allowing direct performance comparison between Barracuda’s interpreted bytecode and native CUDA code. The Barracuda-interpreted version completed computations at an average runtime of approximately ${2.61\,\mathrm{\text {s}}}\pm {0.02\,\mathrm{\text {s}}}$ per iteration, whereas the native CUDA implementation completed iterations in ${0.200\,\mathrm{\text {s}}}\pm {0.004\,\mathrm{\text {s}}}$, resulting in an overhead factor of about 13 (see Fig. 3). For further reference, the same algorithm running on a single CPU thread took ${30.9\,\mathrm{\text {s}}}\pm {0.2\,\mathrm{\text {s}}}$ (approximately 12 times slower than the Barracuda program).

While this performance overhead is notable, it is justified in scenarios where runtime flexibility is required and is expected given the performance difference of other interpreted languages [34]. In biomedical simulations, particularly MRI, dynamic adaptability is often a more necessary requirement than the raw speed benefits of static compilation. It is also important to note that these performance comparisons do not account for the overheads incurred in the embedded-use case. Programs embedding Barracuda should be optimized to follow the no-overhead principle [35], so that any embedding-related performance overheads are only incurred when strictly necessary and only within small regions of the application—thereby minimizing their impact on total runtime.

### MRI simulation examples

Herein, we provide two examples of using Barracuda within our MRI simulation framework. The first example focuses on dynamic $T_1$ changes resulting from the diffusion of a contrast agent. As discussed earlier in Section 2, the Bloch equations were solved using a symmetric Strang splitting approach. In this example, Barracuda routines are embedded within the simulation loop to recalculate $T_1$ values dynamically as per Eq. 2. These recalculations are based on real-time changes in the local tissue characteristics, allowing the simulation to adjust for factors such as local heating or contrast diffusion changes.

**Fig. 4:**
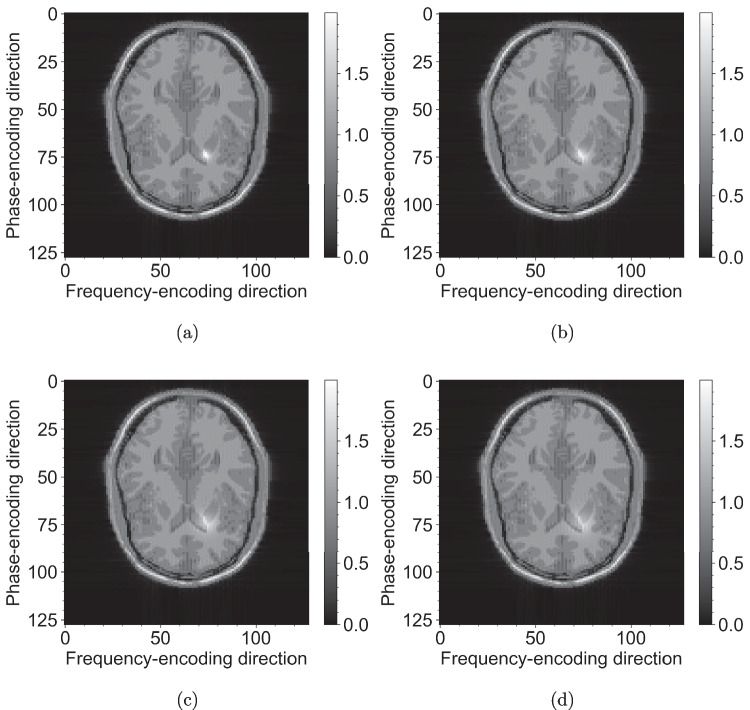
Simulated $T_1$ contrast diffusion images using the McGill brain phantom [38] and a $T_1$-weighted CINE SPGR sequence ($T_E = {8\,\mathrm{\text {m}\text {s}}}$, $T_R={102\,\mathrm{\text {m}\text {s}}}$, zero delay between frames). The propagation term and resulting changes to $T_1$ relaxation times are provided by Eqs. 1 and 2 with $\sigma _0 = 4\, \text {mm}$, $\lambda = 0.0012\, \text {s}^{-1}$, $D=0.1\, \text {mm}\, \text {s}^{-1}$, $A = 0.7$, $x_0=20\, \text {mm}$, $y_0=-20\, \text {mm}$. Images shown correspond to frames 0 (**a**), 10 (**b**), 20 (**c**), and 30 (**d**) of the CINE sequence

**Fig. 5:**
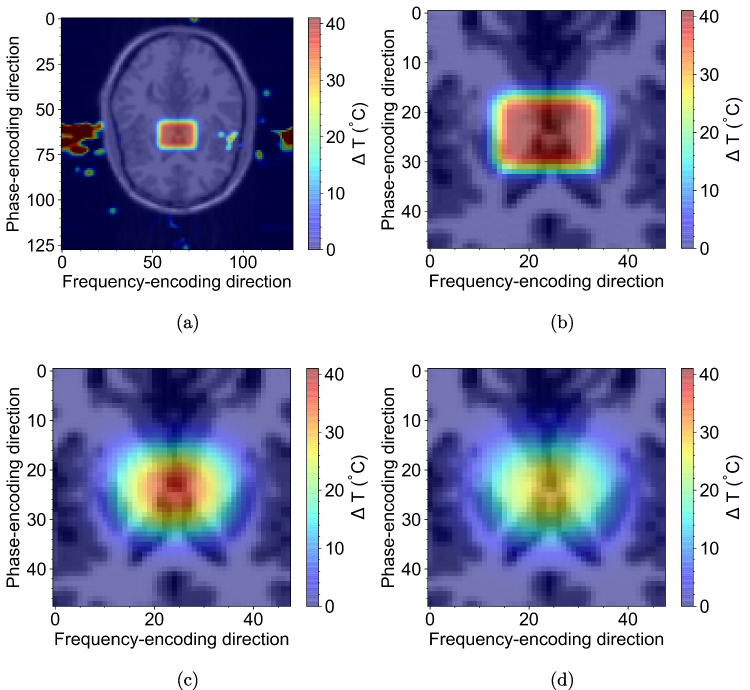
Simulated thermometry images using the McGill brain phantom [38] and a $T_1$-weighted CINE SPGR sequence ($T_E = {8\,\mathrm{\text {m}\text {s}}}$, $T_R = {102\,\mathrm{\text {m}\text {s}}}$, zero delay between frames). A rectangular (${4\,\mathrm{\text {cm}}} \times {3\,\mathrm{\text {cm}}}$) distribution (given by Eq. 3) was used with an initial temperature ($T_0$) of ${40.3\,\mathrm{ ^{\circ }\text {C}}}$ and thermal diffusion coefficients of $k_x = 0.3\, \text {mm}^{2}\, \text {s}^{-1}$ and $k_y = 0.2\, \text {mm}^{2}\, \text {s}^{-1}$. Thermometric images for CINE frame numbers 0 (**a**), 0 (**b**), 10 (**c**), and 20 (**d**) are overlaid on top of magnitude images with ${50\,\mathrm{\%}}$ blending

The simulation of $T_1$ contrast diffusion images generated using the McGill brain phantom [36] in combination with a $T_1$-weighted CINE SPGR sequence [1, 37] (with $T_E = {8\,\mathrm{\text {m}\text {s}}}$ and $T_R = {102\,\mathrm{\text {m}\text {s}}}$, and no delay between frames) is shown in Fig. 4. In this simulation, a propagation model is used to introduce dynamic changes in $T_1$ relaxation times as governed by Eqs. 1 and 2. Key parameters, including $\sigma _0 = 4\, \text {mm}$, $\lambda = 0.0012\, \text {s}^{-1}$, $D = 0.1\, \text {mm}\, \text {s}^{-1}$, and an amplitude $A = 0.7$, with spatial offsets of $x_0 = 20\, \text {mm}$ and $y_0 = -20\, \text {mm}$, define the diffusion process. The resulting images, captured at frames 0, 10, 20, and 30, clearly demonstrate how the $T_1$ contrast evolves over time, capturing the interplay between diffusion-driven propagation and relaxation dynamics. The entire 40 frames took ${504\,\mathrm{\text {s}}} \pm {2\,\mathrm{\text {s}}}$ to simulate using Barracuda. In contrast, a hard-coded CUDA-native solution took ${140\,\mathrm{\text {s}}} \pm {1\,\mathrm{\text {s}}}$, yielding an overhead factor of 3.6. This detailed temporal mapping, along with a smaller-than-theoretical overhead factor, reinforces the utility of Barracuda in the simulation environment for investigating dynamic MR imaging phenomena and understanding subtle tissue contrast variations.

The second example models temperature differences by adjusting the off-resonance frequency ($\Delta \omega$). In MRI, temperature variations within tissue can lead to shifts in the local magnetic field, affecting $\Delta \omega$ and the overall signal. By incorporating Barracuda routines to modify $\Delta \omega$ dynamically, our simulation can capture the impact of temperature differences on the MRI signal.

Figure 5 shows thermometric images using the McGill brain phantom. These images are produced by subtracting the phase at each frame from a baseline image produced with $\Delta \omega _0 = 0$ in order to determine the phase differences for each frame. These phase differences are then converted to temperature differences by using the following relation [9]; $\Delta T = \frac{\Delta \phi }{\alpha \gamma T_E} ,$ where $T_E$ is the pulse sequence echo time. The 40 simulated frames took ${609\,\mathrm{\text {s}}} \pm {3\,\mathrm{\text {s}}}$ to simulate using Barracuda. In contrast, the hard-coded CUDA-native solution too ${162\,\mathrm{\text {s}}} \pm {2\,\mathrm{\text {s}}}$, yielding an overhead factor of approximately 3.8. The dynamic modulation of off-resonance frequency provided a nuanced representation of temperature effects on magnetization evolution and only incurred a 3.8 overhead compared with natively compiled functions, further validating the utility of Barracuda in modeling complex, real-time phenomena.

Averaged NVIDIA Nsight Compute [39] profiles for natively compiled CUDA versions and Barracuda-enabled versions of the MRI simulations are shown in Table 3. Profiles shown are for a single image rather than for all CINE frames, as it was found that simulating all the CINE frames scaled the number of cycles and was therefore unnecessary. These profiles show that our MRI simulation kernels are natively memory-bound; therefore, the increase in memory-boundedness from Barracuda does not have as significant a performance impact as it did on the Mandelbrot example. The Mandelbrot application transitioned from being a purely compute-bound application to a memory-bound application when Barracuda was used as the computations became dependent on reads and writes from memory. Additionally, it seems that there is a small discrepancy between the ratio of average kernel duration (from Table 3) and the overhead factors determined from the total simulation time (4.33 vs 3.6 and 4.39 vs 3.8 for the contrast and thermal diffusion cases, respectively). This is likely due to some differential overhead between the kernels during profiling, and also due to the fact that this profiling measures kernel execution time rather than entire program runtime, thereby ignoring memory allocation overheads that would influence the relative overall performance.

Finally, the number of lines of code and the ratio of these are shown in Table 4. This gives an idea of the ease of use of Barracuda as we can see from the Mandelbrot and Rule 110 cases, a significant reduction in the number of lines of code required due to not requiring the writing of boilerplate kernel-wrapping and handling CUDA code. These ratios do significantly reduce for implementation however (as shown by the contrast and thermal diffusion ratios in Table 4), as the majority of boilerplate code is assumed to have already been written. This overall reduction in code required, along with a simpler syntax (as shown in Listing 1) compared to CUDA, demonstrates the ease of use that Barracuda provides to the end user over native CUDA applications or other C++ implementations, such as Thrust, LLNL RAJA, and Alpaka.

## Discussion

Results from simulations presented demonstrate that Barracuda is a robust and versatile tool capable of bringing dynamic flexibility to high-performance GPU-based simulations. Its minimal yet expressive instruction set, coupled with an efficient high-level language and compiler, allows for real-time modification of simulation parameters without the need for extensive code changes. Although our benchmarks indicate a performance overhead compared to statically compiled CUDA kernels, this trade-off is acceptable in many scenarios, particularly in research and development contexts where adaptability, rapid prototyping, and user customization without directly modifying validated software are essential.

**Table 3:** Processed NVIDIA Nsight Compute profiles for the MRI simulated cases with both natively compiled CUDA code and using Barracuda

	Registers	Cycles	Duration	Compute throughput	Memory throughput
Contrast diffusion - Native	32	20,023,478 (18,592,328–21,471,222)	14.877 ms (12.840–17.250 ms)	55.044 % (52.490–57.960 %)	21.674 % (19.730–23.520 %)
Contrast diffusion - Barracuda	32	83,508,189 (74,172,132–99,044,223)	61.448 ms (52.950–75.280 ms)	22.754 % (18.160–27.760 %)	22.906 % (22.080–23.410 %)
Thermal diffusion - Native	32	23,234,982 (21,328,898–25,500,206)	17.810 ms (15.750–20.660 ms)	59.353 % (56.730–62.100 %)	18.985 % (17.930–19.880 %)
Thermal diffusion - Barracuda	32	109,273,969 (91,187,229–127,342,193)	78.244 ms (64.510–94.610 ms)	17.205 % (13.020–21.420 %)	24.262 % (23.770–24.740 %)

The displayed values are arithmetic means, along with the minimum and maximum values. The register count gives the number of registers per thread and has been optimized for maximum GPU occupancy

**Table 4:** Number of lines of code and the ratio of lines of code (excluding white space and comments, with one assignment per line) between native CUDA solutions and Barracuda

	Mandelbrot	Rule 110	Contrast diffusion	Thermal diffusion
Native	123	146	34	42
Barracuda	32	49	22	28
Ratio	3.84	2.98	1.55	1.50

For the contrast diffusion and thermal diffusion cases, these lines of code are instead additional lines of code required, rather than the complete lines of code for the entire MRI simulator

Due to the authors’ research interests, the application examples focus on MRI simulation; however, Barracuda’s underlying design is general purpose and can be applied to various GPU-based simulations. Regardless of the system being modeled, the ability to dynamically adjust simulation parameters in real-time often provides a significant advantage. Barracuda’s modular design allows it to be incrementally integrated into existing projects, making it an attractive option for researchers across various disciplines who seek to enhance their simulation capabilities with dynamic runtime adaptability.

Future enhancements and compiler optimizations, such as differentiating between static and dynamic arrays, single-input multiple ops, and reducing redundant instructions by compilation-time analysis, could further reduce runtime overhead associated with dynamic interpretation, thereby narrowing the performance gap between interpreted and statically compiled code. Expanding the instruction set to include more domain-specific functions could improve Barracuda’s efficiency and broaden its applicability in specialized fields. Additionally, simulations of more complex phenomena (e.g., fluid flow around realistic vascular structures) [40–42] are interesting areas of further development and testing of the PhoenixMR simulation framework with Barracuda.

Barracuda currently supports GPUs with NVIDIA compute capability 3.5 and higher [43]. Since this is a header-only library that is reasonably lightweight and utilizes CUDA-native functions where possible, it is expected to continue supporting this range of compute capabilities into the future. Where changes to support occur (e.g., due to integrations of newer features), there will be options for disabling these to maintain legacy support for applications that do not wish to narrow their compatibility window.

## Conclusion

Barracuda represents a significant advancement in dynamic GPU-based simulation technology. By combining a carefully designed virtual machine with a high-level programming language and allowing seamless integration into CUDA environments, Barracuda enables real-time modifications of simulation parameters while still achieving high-performance execution. Our experimental evaluation included benchmarks with the Rule 110 cellular automaton, Mandelbrot set computations, and MRI simulation examples demonstrating dynamic $T_1$ adjustment and temperature-induced off-resonance frequency modeling. The results confirm that Barracuda provides both flexibility and practicality for high-performance simulations.

While the design of Barracuda incurs a performance penalty compared to fully compiled CUDA code, Barracuda offers enhanced runtime flexibility, ease of development, and rapid prototyping, making it an attractive tool for GPU-based simulations that can benefit from dynamic adaptability. Future work will focus on optimizing the execution engine and compiler, expanding the VM’s capabilities with additional specialized operations, and incorporating advanced techniques to minimize overhead. In summary, Barracuda successfully bridges the gap between static, high-performance GPU programming and the dynamic requirements of modern simulation environments, providing a robust and extensible platform for researchers across a wide range of disciplines. The code for Barracuda is all open-source and has been released under the GNU GPLv2; there are three public repositories: the Barracuda VM, the Barracuda Compiler, and the Barracuda testing suite. These allow developers of CUDA applications to integrate Barracuda into their libraries quickly, with end-users benefiting from the increased flexibility their simulations are now capable of.

## Appendix A: Rule 110 Barracuda code and bytecode arrays

The following shows both Barracuda code in Listing 1 and the first 50 out of 486 elements of the corresponding generated bytecode arrays (Listings 2–4) produced by the compiler. This is for illustrative purposes to demonstrate the complexity of bytecode and why writing over code in the natural Barracuda language is more mentally challenging.
